# Materia Medica and Therapeutics

**Published:** 1861-09

**Authors:** Emil Fischer


### MATERIA MEDICA AND THERAPEUTICS; MEDICAL CHEMISTRY; TOXICOLOGY AND LEGAL MEDICINE.

BY EMIL FISCHER, M.D.

### MATERIA MEDICA AND THERAPEUTICS.

I.—*On the Employment of Digitalis in Organic Affections of the Heart.* By M. PFAFF.

M. Pfaff recommends the following rules in the administrations of digitalis in disease of the heart: The drug should be administered, not in increasing, but in decreasing doses; and the dose should be diminished as soon as the paralyzing action on the heart and arterial system is perceived. The calmative effect produced by digitalis on the activity of the heart, when morbidly excited, is durable, and is often prolonged for five or eight weeks. The administration of the medicine, in any form whatever, ought not to be continued for more than six or eight days; and if in that time the desired results have not been obtained, recourse should be had to squills or colchicum. These two produce also a calmative action on the heart; and if, after their use, we again have recourse to digitalis, the medicinal symptoms are manifested more promptly, and continue for a greater length of time. In torpid subjects it is advisable to precede the administration of digitalis by a preparatory treatment with squills and colchicum; and in the majority of cases it is of advantage to combine the digitalis with aromatics, bitter extracts, or tonics, in order to avoid the disturbing effects on the digestive organs. With aged persons it is best to give it along with quinine; in tubercular constitutions, united with opium; in dropsical cases, with liquor potassæ, acetate of ammonia, squills, juniper, etc.; in plethoric individuals, it may be associated with cream of tartar, magnesia, sulphate of potash, and nitre; and in the anæmic, with the extract or tincture of malate of iron. By following up the use of digitalis by the administration of arsenic, the cyanotic effects of disease of the heart may often be considerably diminished.

With respect to the different preparations of digitalis, M. Pfaff prefers the infusion to the powder, which is apt to disorder the stomach. The infusion, on the other hand, is more apt to cause colic. The decoction produces the latter effect in a more marked degree, but presents more decided diuretic properties than the other preparations. The alcoholic tincture has the same action as the infusion, but in smaller doses gives rise to phenomena of cerebral congestion, vertigo, etc. The ethereal tincture produces, even more rapidly, the same effects, which, however, disappear as soon as the cardiac and vascular symptoms are developed.

M. Pfaff also recommends the external application of digitalis in cases where complications prevent its internal administration. He has used it with benefit in the form of a mixture of equal parts of chloroform and ethereal or alcoholic tincture of digitalis; ʒij of the mixture are applied by means of a compress three times a day, the compress being retained as long as a burning sensation is felt. The depressing action of the digitalis is manifested at the end of two or three days.—(*Gaz. Méd. de Paris*, June, 1861, *and Edinburgh Med. Journal*, July, 1681.)

II.—*On the Operation of Aniline upon the Animal Body.* By Dr. Schuchardt. (Archiv für Pathologische Anatomie und Physiologie und für Klinische Medicin, 1861.)

Dr. Schuchardt made a series of experiments to prove the effects of aniline upon animals. Aniline is an oily liquid, of powerfully basic properties, obtained by the distillation of indigo, and the name aniline is derived from the specific name of the indigo plant, *indigofera anil.* The physiological properties of aniline are but little known, but the researches of Dr. Schuchardt prove that it is a poisonous substance. The results at which he arrived are the following:—

Aniline may act injuriously on the animal organism, and in large doses may even cause death. Frogs introduced into a weak solution containing aniline, died in periods varying from a quarter of an hour to two hours and a half; and death was also caused by the introduction of aniline into the mouth or into a wound in the back. Rabbits were also poisoned by this substance, a small animal being killed by fifty drops in six hours and a quarter, and a larger one by one hundred drops in four hours. In all the animals experimented upon, violent clonic and tonic spasms ensued after the application of the aniline, and continued almost uninterruptedly until death. There was also loss of sensibility, commencing at the lower extremities, and extending to the upper; and the temperature of the body was also reduced. Wherever the aniline was applied locally, as in a wound of the back, on the stomach, on the posterior part of the tongue, or on the conjunctiva, appearances of irritation were observed, which are probably connected with the power possessed by aniline of coagulating albumen. The aniline was never detected in the urine, and it is probable that this substance is eliminated from the body rather by the organs of respiration than by the kidneys.—(*British and Foreign Med.-Chir. Rev.*, July, 1861.)

III.—*On the Physiological Action of Santonine and of the Essential Oil of the Seeds of Artemisia Contra.* By Dr. Edmund Rose, of Berlin. (Archiv für Pathologische Anatomie und Physiologie und für Klinische Medicin, 1860.)

Santonine is an organic principle obtained from the artemisia contra, and possesses vermifuge properties; but its operation has only recently been minutely studied. Dr. Rose has taken it himself, in the dose of a gramme, (about fifteen grains,) without injury. Its taste is bitter, very disagreeable and persistent, and it is slightly diuretic, but rather confines the bowels. It produces a peculiar and indescribable narcotic effect, and causes a species of color blindness, tinging all the objects seen with a yellow hue. The essential oil of artemisia contra is poisonous. It kills rabbits in the dose of about two grammes, producing convulsions, which descend from the head to the lower extremities, followed by ascending paralysis. This oil is not vermifuge; for in medicine and medicinal doses it is absorbed in the stomach and the upper part of the small intestines, and does not reach the lumbrici, which generally exist lower down. In larger doses it is poisonous. Santonine, on the other hand, is a good vermifuge. It is innocuous, and traverses the whole of the digestive canal, and is found in large quantity in the fecal matters; for it is almost insoluble in water, and a small part only is transformed into soluble santonate of soda, after contact with the bile, and it is santonine which most rapidly destroys the worms. Santonate of soda ought not to be substituted for santonine, as has been sometimes recommended, for it possesses no superiority over the latter, and is, besides, of a more disagreeable taste, and is poisonous in large doses.—(*British and Foreign Med.-Chir. Rev.*, July, 1861.)

IV.—*On the Local Action of Savin.* By DR. EISENMANN, of Wurzburg.

The Hungarian Journal of Physiology and Medicine, 1859, No. 5, and from it the Medico-Chirurgical Monthly Papers for May, 1859, contain a communication by Dr. Moller entitled "Savin as a Popular Remedy," according to which it would appear that in Hungary a decoction of savin is in common use for the removal of polypi. The author is acquainted with the case of a peasant woman, who by means of this agent got rid of a nasal polypus; and he himself successfully employed it to destroy and remove a soft nasal polypus which he had twice in vain extracted. On account of the softness of the polypus, he had not been able to extract the root, and it had consequently always again grown rapidly. The following is the mode of using it:—

A drachm of savin-tops was boiled for five minutes, in about three or four ounces of water, and the polypus was painted frequently during the day with the filtered decoction; the fluid was also injected into the nose.

This communication reminds me of some results I have myself observed from savin. In Hecker's old Materia Medica, the following preparation is found under the name of Hecker's salve:—

An onion is roasted in the ashes; the juice is expressed, and with this juice, and a corresponding quantity of powdered savin, a sort of liniment is prepared. This liniment is recommended by Hecker in the treatment of condylomata, and in fact it removes venereal warts in a suprisingly short time. During twenty years' experience I repeatedly observed very fully developed condylomata disappear completely after three days' use of this ointment. These remarks may serve to introduce the following:—

During my stay at the fortress of Oberhaus, at Passau, I became acquainted with Bonkratz, the drummer of the battalion, who attracted my attention in consequence of a tumor he had on his neck, beneath the right ear. This tumor was circular and flat, was equal in circumference to a crown-piece, rather over than under it, and was about a quarter of an inch in thickness. It had a tolerably wide base, was hard as fibro-cartilage, and presented a bluish-red appearance. This tumor had been frequently removed with the knife, and its root extirpated by the actual cautery, but it had always grown quickly again. In other respects Bonkratz was in good health, had no traces of syphilis, and his skin was free from eruptions and ulcers. The causes of the tumor were quite unknown. I spoke to Dr. Delzer, at that time surgeon to the regiment, about the case; mentioned my observations on the efficacy of savin in the treatment of condylomata, and suggested to him to make a trial of Hecker's salve with this swelling, which resisted all other remedies. Dr. Delzer, whom I always found extremely friendly, agreed to my proposal, and promised to carry it out. However, in September, 1843, I left the fortress, and lost sight and recollection of Bonkratz. In the autumn of 1847 I met the same Bonkratz at Nurnburg, where he had been employed at the custom-house, quite unexpectedly in the street. The tumor in his neck was completely gone, and, as he said, in consequence of the use of the remedy I had recommended.

Savin, therefore, exercises a remarkably deletary influence on condylomata, polypi, and tumors, which may perhaps be regarded as polypi of the external skin; and who knows how much farther this influence may extend?—(*Virchow's Archiv*, Band xviii., 1 und 2 Heft, p. 171; *Dublin Quarterly Journ. of Med. Science*, May, 1861.)

---

### MEDICAL CHEMISTRY.

I.—*Formation of Glucose with Chondrine.* By MM. FISCHER and BŒDECKER.

The costal cartilages of six dead bodies were at first cleared of the tendons, then washed in hydrochloric acid, and finally boiled with this acid in a concentrated state. When, after neutralization by an alkali, the liquid reduced the cuprotartric solution, the quantity of sugar produced was determined.

The authors have found by direct experiments that the chondrine employed as aliment augmented the proportion of urea and of sugar in the urine; the density of the urine was not sensibly affected, nor the quantity secreted. The urine was constantly acid. The experiments lasted six days. During three days a normal regimen was followed, of which, however, sugar and chondrine were excluded; during the three other days 500 gr. of the latter substance, in the state of compact jelly, was taken, seasoned with 6 gr., 25 of sea salt; the jelly represented 36 gr. of chondrine dried at 120°.

Under the influence of this regimen the urea increased from 32 gr. to 41; the sugar from O gr., 407 to O, 509 and 847.—(*Ann. der Chem. und Pharm.*, t. cxvii. p. 111, 1861.)

II.—*On Alcaptone, immediate Principle of a Pathological Urine.* By MM. BŒDECKER and DURR.

This substance was found in the urine of an asthmatic patient, who had previously been affected with typhus. Its presence is easily proved by the action of potassa; the urine became rapidly brown on absorbing oxygen from the air. The alcaptone, as M. Bœdecker calls it, reduces the cuprotartric liquid, but it has no effect upon the hydrate of bismuth.

In order to extract it, the urine is first precipitated by the neutral acetate of lead, then, after filtration, by the tribasic acetate, of which an excess must be avoided; the precipitate, which contains all the alcaptone, is washed in water, then decomposed by sulphuretted hydrogen. After evaporation in the water-bath it is redissolved in ether and left to crystallize; some crystals of hippuric acid are deposited, the brownish-red residue gets dry and furnishes a vitreous mass, which becomes viscid, at the air, is fusible, inflammable, and decomposes itself, giving off an offensive odor, which reminds one of the principles of bile.

The alcaptone is soluble in water and in alcohol; pure ether is without action. It contains nitrogen.

On applying heat, it reduces the nitrate of silver and of mercury, chromic acid and permanganic acid. It does not ferment.

By its behavior in regard to the cuprotartric liquid and to hydrate of bismuth, it approaches uric acid.

In the urine which contained this substance, glucose was constantly present. It always differed from diabetic urine in the fact that the proportion daily secreted did not exceed 1500 C.C., and never contained more than 1 per cent. of sugar.—(*Annal. der Chem. und Pharm.*, t. cxvii. p. 98, 1861.)

---

### TOXICOLOGY AND LEGAL MEDICINE.

### *On the Detection of Arsenic.* By DR. TAYLOR.

In an elaborate paper in the last volume of the *Guy's Hospital Reports*, (third series, vol. vi.,) entitled "Facts and Fallacies connected with the research for Arsenic and Antimony, with suggestions for a method of separating these poisons from organic matter," Dr. Taylor passes in review the processes of Marsh and Reinsch, giving many useful hints as to their proper performance, and pointing out the fallacies against which the analyst must be on his guard, and especially noting that fallacy which gave such notoriety to the Smethurst trial. As to Marsh's process Dr. Taylor gives the following estimate, p. 207: "It may be stated that there are two difficulties which the analyst encounters in resorting to it for the separation of arsenic from the solids or fluids of the body. First, there is no good or simple method of bringing the arsenic to a concentrated state, *i.e.* to a state fitted for testing, without producing froth; and secondly, when the quantity of arsenic in an organic liquid is so small as to render the adoption of the process necessary, it is not possible to follow the plan of the inventor, of generating the hydrogen in the whole quantity of the organic liquid."

*Reinsch's Process.*—The process of Reinsch is now rendered as satisfactory for the detection of antimony as of arsenic; and by comparison with the process of Marsh, it is not only more delicate, but far more convenient in its application. Marsh's process may be used simply for corroboration.

In comparing the two processes for the separation of antimony from liquids, I have found that Reinsch's method is twenty times more delicate in revealing the presence of antimony than that of Marsh, as it is usually employed.

There is this difference between the two discoverers, Marsh and Reinsch—

while the former suggested a useful application of facts already made known by others, the latter discovered for himself the simple fact upon which his process is based. Until within a recent period it was thought too, that the materials used in the process of Reinsch were far less liable to give rise to fallacious results than the materials required for the process of Marsh. The investigations, however, which were carried out by Dr. Odling and myself in the case of Isabella Banks, (the case of The Queen against Smethurst, August, 1859,) showed that there was a latent fallacy connected with the use of Reinsch's process, of the existence of which neither Reinsch nor any toxicologist of repute had up to that date the slightest suspicion. This was the discovery of arsenic in all the best and so-called purest varieties of copper, whether as foil, gauze, or wire, and in sufficient proportion to give rise, under certain conditions, to fallacious results.

*Arsenic as an impurity in Copper.*—It has been hitherto considered that one of the great advantages of Reinsch's process was, that while hydrochloric acid might easily be obtained pure, no question could arise about the impurity of copper. The presence of arsenic in the purer forms of this metal was, until 1859, either not suspected or wholly ignored by chemists of the highest authority. It now turns out that there is no kind of copper available for use which is free from arsenic; and some kinds of foil and wire hitherto used and still employed by analysts of repute contain arsenic united to copper as arsenide in comparatively large proportion. In fact, whether among English, or French and German writers on chemistry and toxicology, there is not one who has ever pointed out, or even suspected, that this process would lead to error by reason of the universal presence of arsenic in copper, commercial or refined, which is accessible to the chemist. All have hitherto relied upon the cleanness of the surface of the copper used, and the method of testing the acid and copper first pointed out by Reinsch. As a general rule, this is quite sufficient. There is no liability to fallacy unless the copper is used on a large scale, and there is an acid or salt present which acts upon and dissolves the impure metal. Pending its solution the atoms of arsenic combined with it as arsenide or arseniuret are set free, and may thus attach themselves to the clean surface of the undissolved copper. It is clear that if the presence of a minute quantity of arsenic in copper presents that great risk of fallacy which some chemists have recently endeavored to impress on the public mind, no one of the numerous analyses for arsenic by Reinsch's process, which have been undertaken during the last twenty years, could have been made without the inevitable discovery of arsenic. In short, if a source of fallacy, it would have led to the constant and inevitable discovery of arsenic in every solid and liquid submitted to examination. Analysts who have had much experience in the use of this process, will bear me out in the statement that, in employing samples of the same copper and acid, for one affirmative result in which arsenic is discovered there will probably be four or five negative results, in which no deposit whatever has taken place on the arsenical copper and no arsenic was detected. By boiling successive quantities of the same arsenicated copper in the same acid liquid containing arsenic, the whole of the free arsenic is finally removed; the deposits on the metal become less and less decided, they at length cease altogether, and the last portion of copper put into the liquid comes out nearly as bright as when introduced, or if at all dull on the surface, this dullness arises not from arsenic but from oxidation, as a result of long boiling, It is obvious that if the arsenic came from the copper itself and not from any extraneous source, instead of the arsenic diminishing on each introduction of the copper, it would go on increasing in proportion to the quantity of the metal employed in the analysis. The hundreds of negative results which have been obtained by experienced analysts establish the untruthfulness of the assertion that the process, as it is commonly employed, is attended with a serious risk of fallacy, even when copper containing arsenic is unknowingly used. The analysis performed by Reinsch, Gaultier de Claubry, Orfila, Christison, Maclagan, Geoghegan, Watson, Rey, Penny, and others, are as unassailable on this ground as those which have been performed by Mr. Herapath himself, even with his No. 13 commercial wire, *i.e.* assuming that the copper and acid have been boiled together and no deposit formed, before the suspected substance was added.

Is there any risk in employing copper containing arsenic for separating this substance in cases of poisoning? The answer may be anticipated. There is no risk unless the copper during the process is brought to a state of solution; and of this fact the operator is immediately informed by the disappearance of the metal, and by the liquid acquiring a deep-green color. Hence, while the negative results which the process so frequently furnishes afford an unquestionable proof that the copper may be used with safety, the operator has clear evidence when there is risk, by the solution of the copper in the acid liquid. If the copper employed is unaltered in weight and is not to any perceptible extent dissolved, it is obvious that the liquid submitted to analysis cannot have received any impregnation of arsenic from the copper employed, and if, under these circumstances, arsenic is deposited on the copper, then that poison must have been present in the liquid submitted to examination. Cold hydrochloric acid, if strong and not too freely exposed to air, produces no deposit or change in the appearance of bright copper even after forty-eight hours' immersion and exposure. Whether the copper be pure or arsenicated, there is no perceptible difference. The upper stratum of the liquid has a slight, greenish-yellow tint, arising from the oxidation and solution of a small quantity of copper. If the polished copper is immersed in an acid diluted with eight parts of water, it will be found after forty-eight hours that the whole of the liquid has a greenish color, from the formation of chloride; the copper, if arsenicated, is coated with a thick, dark-colored deposit, while if pure, it is of a reddish-brown color. The surface of the metal in either case will be found much corroded, and a number of tetrahedral crystals of sub-chloride of copper are scattered over it. I have not been able to procure crystals of arsenious acid by heating impure copper thus coated, and I have not detected any arsenic in the acid liquid. The amount of arsenicated copper dissolved is probably too small to furnish any evidence of arsenic. These results confirm Reinsch's statement, that the concentrated acid, *cœteris paribus*, exerts a less powerful action on copper than the diluted acid; they also prove that arsenicated copper should not be allowed to remain for a long time in the acid liquid. The tarnish thus formed might, unless properly tested, be mistaken for arsenic, or conceal an arsenical deposit. They prove, however, that the alloy of arsenic and copper (arsenide) is not so decomposed by cold, diluted hydrochloric acid as to set free any arsenious acid in the liquid. In short, the danger of any fallacy in the ordinary use of the process, arises from the presence of arsenic as an impurity in the acid and not from arsenic contained as impurity in the copper. But for this fact no chemist would ever have met with negative results in employing copper in the state in which it has hitherto been used.

Hydrochloric acid diluted with from six to ten parts of water has but a feeble action on copper, even after long boiling. A trace of this metal may under these circumstances be found in the liquid. If, however, the oxide of arsenic or antimony should be present in the liquid, a portion of copper is immediately dissolved and an equivalent portion of arsenic or antimony is deposited. So soon as the copper is completely coated, the solvent action is arrested.

In order to determine how far arsenicated copper is liable to undergo solution and to set free arsenic as a result of boiling in pure hydrochloric acid, diluted, the following experiment was performed: a quantity of copper foil and gauze, equivalent to 39·4 grains in weight, was boiled in a retort with a mixture of one ounce of pure hydrochloric acid and eight ounces of water. The boiling was continued for half an hour, and the distilled acid liquid was collected in a cool receiver. This amounted to twelve fluidrachms; it contained no trace of copper; and on a careful examination of the liquid by Marsh's and Reinsch's processes, there was not the slightest trace of arsenic in it. The large quantity of acid liquid left in the retort was found to contain a small quantity of copper, but there was no arsenic dissolved in it. The undissolved copper, when washed and examined by the microscope, had no metallic deposit upon it. It was unchanged in color, but presented slight marks of corrosion; a quantity of it heated in a tube gave no sublimate.

When arsenicated copper is boiled in the diluted acid for five minutes—a period within which arsenic, if present, is commonly manifested by some deposit or change of color on the surface of the metal—no copper is dissolved. The metal retains its usual polish and lustre. If a small portion of oxide of arsenic or antimony be now added to the boiling acid liquid, there is an immediate deposit on the clean copper surface, and copper in small quantity will be found dissolved in the liquid by that delicate test—ferrocyanide of potassium. It is therefore obvious that the metallic oxide (*i.e.* the poison itself) brings about the solution of the copper, but not to an extent to eliminate arsenic so as to affect the process. It has been alleged that the accidental presence of certain salts, or organic liquids not containing arsenic, would lead to a sufficient solution of the copper to give rise to a deposit of arsenic on that metal. This action has been loosely ascribed to alkaline nitrates, phosphates, sulphates, and chlorides, salts which may be occasionally met with in organic liquids. A grain of each of these salts was dissolved in two drachms of diluted hydrochloric acid, in the proportions used for the precipitation of arsenic, (1·8.) The solution was brought to the boiling point and boiled for a few minutes in contact with a slip of polished copper. No copper was dissolved in any one instance, and the metal retained its lustre. On adding a minute portion of arsenic to the liquid, the metallic copper was speedily coated, and traces of a salt of copper were now for the first time found in the liquid. When to a similar quantity of acid liquid in which copper was immersed, a fragment of chlorate of potash was added, there was an immediate solution of a portion of the metal, a fact rendered evident by the production of a green color and by the application of the usual tests for copper to the liquid.

While the presence of a sulphate, nitrate, chloride, or phosphate does not affect the process, so long as a properly diluted acid is employed for a proper time, it was found that an alkaline chlorate, even in small quantity, caused a rapid solution of the copper, and in proportion to the amount of this solvent action, a liberation of arsenic from the arsenicated copper. Owing to this chemical action of a chlorate on copper, it has been contended that Reinsch's process ought never to be employed when such a salt is present. Mr. Herapath affirms that "Reinsch's process is not applicable where nitrates or chlorates are present." With respect to the nitrates, it admits of distinct proof that they present no objection when a properly diluted acid is used; and with regard to the chlorate, the process is unobjectionable, provided pure copper be used. The operator, in using copper free from arsenic, has only to saturate the liquid with the metal, and the last portion of copper put into it will effectually remove the whole of the arsenic, as well as any antimony that may be present. It is a good method of detecting minute traces of arsenic or antimony in copper, and one free from all objection, provided the hydrochloric and the chlorate are pure. The only circumstance which would render Reinsch's process inapplicable to the separation of arsenic in the presence of a chlorate, is the use of copper containing arsenic, like the No. 13 wire, or the dial plate or foil, as it is commonly sold. It is only the analyst who has habitually and knowingly used arsenicated copper who can treat the presence of a chlorate in an organic liquid as a serious obstacle to the employment of this process.

While, however, there is no reason to believe that the use of ordinary copper is attended with any risk in the employment of Reinsch's process, merely because it contains traces of arsenic in intimate chemical union, it may be a question whether, out of deference to public opinion, a substance containing arsenic in any form or in any proportion, should be used for the detection and separation of this poison. In several medico-legal cases which have occurred since the universal existence of this impurity has been made known, the ordinary copper in gauze and foil has been employed by gentlemen who, at the time, believed they were using pure copper; they have relied for purity only on the negative mode of testing the metal suggested by Reinsch. A chemist who does this must always incur some risk; and the result may be that a jury will be induced to reject his evidence, simply because he has employed an article actually containing the poison for which he was seeking.

Whatever explanation he may give as to the non-solution of the copper, and the proportion of arsenic found being much greater than any arsenic contained in the copper which he used could possibly account for, it may always be suggested that he actually put the arsenic into the liquid. This equally applies to the use of the impure metal for the detection of antimony, as antimony is not an uncommon impurity in copper. To meet this objection it will therefore be absolutely necessary in future to seek for copper free from either arsenic or antimony.

*Methods of detecting Arsenic in Copper.*—There has hitherto been great difficulty in detecting small quantities of arsenic in copper; and this may account for the fact that samples of copper are sold as pure, and are pronounced to be free from arsenic, when a more careful research would have demonstrated the presence of this substance. This difficulty may be appreciated from the fact that when samples of the arsenicated copper gauze used in the Smethurst case were forwarded to five experienced analysts in England, Scotland, and Ireland, three succeeded and two failed in detecting arsenic in it. A sample of Burra-Burra copper, supplied to the Royal Mint as absolutely free from arsenic, was found (by a process to be presently explained) to contain traces of that substance.

Some Swedish copper, which had been reserved as pure by a distinguished chemist, was found on analysis to be arsenical. The detection of the arsenic depends entirely on the method pursued.

The first plan which I adopted successfully, consisted in oxidizing the copper by nitric acid, evaporating the acid liquid to dryness, and then heating the residue to procure the black oxide, and at the same time to convert the arsenic, if present, to arsenic acid. This acid was subsequently detected in the residue by digestion in water and the addition of nitrate of silver.

Another plan, proposed and employed by Dr. Odling, clearly showed the presence of arsenic in a small quantity of gauze, foil, or wire. The copper was entirely dissolved by the aid of hydrochloric acid and a weak solution of chlorate of potash. The surplus chlorine was removed by the gradual addition of small quantities of bisulphate of soda, and the sulphurous acid derived from this salt was removed by boiling—the freedom of the vapor from the acid being indicated by holding in the current starch paper saturated with iodic acid. Reinsch's process was then applied to the liquid, and if the copper under examination contained arsenic, a deposit of arsenic was obtained in the usual way on a piece of copper.

There is no chemical objection to this method; but seeing that several substances are employed in the analysis, and that a mixture of salts resulted, it appeared to me preferable to convert the copper to a soluble chloride by digesting it in pure hydrochloric acid alone, and then from the known volatility of chloride of arsenic, to separate this from the chloride of copper by distillation. For this purpose I found it necessary to expose the copper in a stratum of pure and concentrated hydrochloric acid, contained in a saucer, the metal being partly immersed in the acid and partly exposed to the air. After two or three days' exposure, according to the thickness of the metal, a brown liquid (a solution of subchloride of copper in hydrochloric acid) was obtained. In some experiments the copper was entirely dissolved; in others a portion was left. The weight of the residue, after washing and drying, deducted from the original weight employed, gave the amount of copper dissolved. The brown liquid was distilled to dryness in a flask or small retort, placed in a sand-bath, and the vapor was condensed in a flask or receiver containing a small quantity of distilled water. A perfectly clear solution of chloride of arsenic, or a mixture of hydrochloric and arsenious acids, was thus procured. When one part of this distillate was diluted with its volume of water and boiled, and a small piece of pure polished copper was introduced, the presence of arsenic was speedily indicated by the characteristic deposit on the bright metal. Another portion of the liquid was introduced into a Marsh's apparatus, or a small flask with a bent tube fixed in its neck, and, by the addition of pure zinc, arseniuretted hydrogen was easily procured from it and tested. When to a third portion a solution of chloride of gold was added, and the mixture boiled, the gold was, after a time, completely deposited. The weight of gold thus deposited supplies one method of calculating the quantity of arsenic present in the distillate.

Of the delicacy of this process for detecting arsenic there will be, I think, no doubt on the part of those who try it; and it has this important advantage over other methods, that excepting the copper, the purity of which is to be tested, and hydrochloric acid, the purity of which may be easily determined by a preliminary experiment, no chemical is used. It is equally applicable to the detection of minute traces of arsenic in the sulphate and all the salts of copper, soluble or insoluble. It is only necessary to distill them with a sufficient quantity of pure hydrochloric acid. I had in vain sought for evidence of arsenic in some sulphate of copper by the use of Marsh's process; but on distilling only ten grains of the crystals with hydrochloric acid, chloride of arsenic was obtained in the receiver, and the presence of the poison was satisfactorily proved. In the course of the Smethurst analysis, in July, 1859, Mr. Brande and Dr. Odling adopted, at my suggestion, this method of analyzing some sulphate of copper pills for arsenic, and they both expressed their satisfaction with it as a safe and delicate process.—(*Edinburgh Medical Journal*, May, 1861.)

